# Immunohistochemical Expression of CD_56_ and ALDH_1 _in Common Salivary Gland Tumors

**Published:** 2016-11

**Authors:** Safoura Seifi, Maryam Seyedmajidi, Jahanshah Salehinejad, Hemmat Gholinia, Fatemeh Aliakbarpour

**Affiliations:** 1 *Department of Oral and Maxillofacial Pathology, School of Dentistry, Babol University of Medical Sciences, Babol, Iran.*; 2 *Dental Research Centre, School of Dentistry, Mashhad University of Medical Sciences, Mashhad, Iran.*; 3 *Health Research Institute, Babol University of Medical Science, Mazandaran, Iran.*

**Keywords:** Adenoid cystic carcinoma, ALDH_1_, CD_56_, Immunohistochemistry, Mucoepidermoid carcinoma, Pleomorphic adenoma

## Abstract

**Introduction:**

Natural killer (NK) cells, of which CD_56_ is a specific marker, play an important role in host defense against tumors. Cancer stem cells, of which aldehyde dehydrogenase isoform 1 (ALDH_1_) is an immunohistochemical marker, are a group of tumorigenic cells which are involved in migration and tumor recurrences. We aimed to evaluate the expression of ALDH_1_ and CD_56_ in common salivary gland tumors, as well as their relationship with each other and with a number of clinicopathologic factors.

**Materials and Methods:**

Forty-five paraffin blocks of salivary gland tumors (pleomorphic adenoma, mucoepidermoid carcinoma and adenoid cystic carcinoma, 15 samples each) were selected. Malignant tumors were classified into two groups: low-grade (including mucoepidermoid carcinoma grade I) and high-grade (including mucoepidermoid carcinoma grade III and adenoid cystic carcinoma). Immunohistochemical staining for ALDH_1 _and CD_56_ markers was performed. Data were analyzed using SPSS (20) and the Chi-square test.

**Results:**

CD_56 _expression was significantly higher in benign and high-grade malignant tumors (P=0.01). ALDH_1_ overexpressed in all three salivary tumors, but not to statistically significant degree (P=0.54). There was no statistically significant correlation between ALDH_1_ and CD_56_ expression with demographic factors (age, gender, or location of tumor; P>0.05).

**Conclusion:**

It appears that the number of NK cells and their function change in different types of salivary gland tumors (benign/malignant) and stroma. NK cells are important components of the anti-tumor system; therefore immune dysfunction is associated with tumor progression in tumors of the salivary gland. ALDH_1_ overexpression suggests its role in tumorogenesis, but ALDH_1_ is not involved in the morphogenesis of salivary gland tumors.

## Introduction

Salivary gland tumors are neoplasms with low prevalence and heterogeneous morphology in the jaw and oral region, and account for 3% of head and neck tumors. Pleomorphic adenoma is the most common salivary gland tumor, with 90% of cases occurring in the parotid. Mucoepidermoid carcinoma and adenoid cystic carcinoma are the most common malignant salivary gland tumors, and both show aggressive behavior and a tendency to metastasize. Due to differences in grades of mucoepidermoid carcinoma (low, intermediate, and high grade) and different histopathologic types of adenoid cystic carcinoma (including solid, cribriform, and tubular patterns), these tumors demonstrate varying biological behaviors (1,2).

Natural killer (NK) cells, part of the innate immune system, play important roles in the host defense against tumors. NK cells control different kinds of tumors by restricting their progress and inhibiting infiltration. This function is achieved by direct cell cytotoxicity without previous sensitivity and immune cytokine secretion such as IFNɣ (3,4). NK cells demonstrate CD_56_ and CD_45 _adhesion markers, along with the absence of T-cell receptor (TCR) CD_53_. These cells originate from the CD_34_^+ ^progenitor cells in the bone marrow, and migrate to the lymphoid tissues and peripheral blood for differentiation. Interleukin (IL)-15 is necessary for the development of these cells, while other factors such as TGF-β1 and indoleamine 2,3-dioxygenase 1 (IDO_1_) inhibit their function (5). CD_56_+ NK cells, which constitute the majority of circulating NK cells, are the most potent cytotoxic NK cells against tumor cells (3).

The hypothesis that cancer arises from stem cells was suggested about 150 years ago, but it is only recently, thanks to advances in stem cell biology, that the necessary experimental framework to test the hypothesis has emerged. In the cancer stem cell model, tumors are constructed by tissue stem cells as well as progenitor cells, which have self-renewal features. The empirical evidence for the cancer stem cell hypothesis was provided by Dick in human myeloid leukemia, showing that tumor cells are derived from a small population of cells. This was then extended to solid tumors by Clark and colleagues (6,7). To identify cancer stem cells, different markers such as CD_34_, CD_133_, CD_24_, aldehyde dehydrogenize 1 (ALDH_1_), and platelet endothelial cell adhesion molecule (PECAM) are used (8).

ALDH_1_ is a detoxification polymorphic enzyme, which is responsible for the intracellular oxidation of aldehydes. ALDH_1_ has an important role in the primary differentiation of normal and malignant stem cells through the conversion of retinol to retinoic acid. This enzyme is present in the liver and is metabolized by striated and cardiac muscle. High expression of ALDH_1_ has been reported in normal and malignant stem cells (hematopoietic and central nervous system) and also lesions such as multiple myeloma, leukemia, breast carcinoma, and pancreatic adenocarcinoma. Cancer stem cells have features such as asymmetrical self-renewal, expression of active telomerase, increased permeability of the membrane, the ability to migrate and metastasize, and anti-apoptotic activity (9).

Because of the paradoxical and conflicting results of ALDH1 and CD56 expression in different neoplasms and the lack of an accurate assessment of them in salivary gland tumors, we aimed to assess the ALDH1 and CD56 expression in common benign and malignant salivary gland tumors, as well as their relation with each other and with a number of clinicopathologic factors.

## Materials and Methods

In this cross-sectional descriptive-analytic study, 45 paraffin blocks (15 benign pleomorphic adenoma, 15 mucoepidermoid carcinoma, and 15 malignant adenoid cystic carcinoma) from Babol and Mashhad School of Dentistry archive files were selected. Clinical information, including age, gender, and the place of lesion were recorded. To confirm the histopathologic diagnosis and selection of appropriate block, as well as the grade of mucoepidermoid carcinoma differentiation and histopathologic patterns of adenoid cystic carcinoma, paraffin blocks were cut into 5-micron sections for hematoxylin-eosin staining.

The grade of differentiation of mucoepidermoid carcinoma samples was determined on the basis of Brandwein criteria (10). Then, malignant neoplasms were divided into two groups based on their biologic behavior: a low-grade malignant group consisting of mucoepidermoid carcinoma grade I, and a high-grade malignant group consisting of mucoepidermoid carcinoma grade III and adenoid cystic carcinoma (11). Adenoid cystic carcinoma samples were classified into three groups: solid, tubular, and cribriform, in accordance with the system devised by Neville and colleagues (2).

For immunohistochemical staining of paraffin blocks, 4-micron slices were prepared, and then deparaffinized in xylene and dehydrated in different degrees of alcohol. We used hydrogen peroxide 0.03%, and phosphate-buffered saline (PBS) for washing. Because of antigen retrieval, slides were placed in the microwave (Panasonic 1280 Watt) for 30 min at 120°C and a pressure of 2 atm, then placed at room temperature for 20 min. After washing in PBS, we used superblock solution for 30 min, and the slides were then washed with PBS. 

Primary antibodies, anti-CD56 (Biocare-USA) and anti-ALDH1A1 (Biocare-USA) were applied at room temperature for 1 hour, and washed again with PBS. Next, we use polyvalent solution for 10 min, and then washed with PBS. Finally, horseradish peroxidase (HRP) (ScyTek-USA) was used and again washed with PBS, then3, 3 -diaminobenzidine (DAB) chromogenic (ScyTek-USA) was added for 15 min.

The positive control for evaluation of ALDH1 and CD56 expression was breast ductal carcinoma and oral normal mucosa around the hyperkeratosis area, respectively. Furthermore, rat non-immunized serum was utilized as the primary antibody for the negative control. Brown staining with ALDH1 and CD56 in cytoplasm of tumor cells was considered positive.

To assess NK cell presence in tumors and CD56 expression, the percentage of cells that stained in five microscopic fields with the greatest number of NK cells (hot spot) in ×40 magnification were counted. If more than 10% of cells were stained with the CD56 marker, they were considered positive (12).

In terms of assessment of the ALDH1 expression, the percentage of stained cells as well as the intensity of cytoplasmic staining were considered. Intensity of the tumor cells stained with ALDH1 was recorded in four different degrees (I), consisting of: lack of staining, weak , moderate, and intense staining (0-3). Percentage of tumor cell staining (p) in five microscopic fields with ×40 magnification or the highest number of tumor cells was also evaluated. Degree of staining percentage was based on: 0 (if there was no staining of tumor cells), 1 (staining in ≤1% of cells), 2 (staining in 2–10% of cells), 3 (staining in 11–33% of cells), 4 (staining in 34–66% of cells), 5 (staining in >67% of cells). The total score was calculated based on the sum of I and P. If the total score was 1 or 2, then ALDH1 was considered negative, while a total score of 3–8 was considered to show positive ALDH1 (13).

Data were analyzed using statistical software SPSS (20) and a Chi-square test (χ2). P<0.05 was considered statistically significant.

## Results

The study included 45 blocks of paraffin, consisting of salivary pleomorphic adenoma (15 cases), mucoepidermoid carcinoma (15 cases) and adenoid cystic carcinoma (15 cases). The clinical characteristics of the patients (age, gender, and location of the lesion) is summarized in Table 1.

**Table 1:** Average age, gender and location of lesions in common benign and malignant salivary gland tumors

**Salivary gland tumor**		**Number**	**gender**		**Age mean**	**Location of tumor**	
			Female	Male		Minor salivary gland	Major salivary gland
Pleomorphic adenoma		15	10	5	44.2±9.51	8	7
Low-grade malignant tumors (Mucoepidermoid carcinoma grade I)		8	5	3	43.12±13.84	1	7
high-grade malignant tumors	Mucoepidermoid carcinoma grade III	7	4	3	55.71±13.01	1	6
	Adenoid cystic carcinoma	15	7	8	48.26±14.39	4	11

*H&E staining:*

From 15 cases of mucoepidermoid carcinoma, eight were of grade I and 7 were of grade III. From 15 cases of adenoid cystic carcinoma, based on their histopathologic pattern, three were cribriform, seven were tubular, and five had a solid pattern.

*Immunohistochemical:*

**Fig1:**
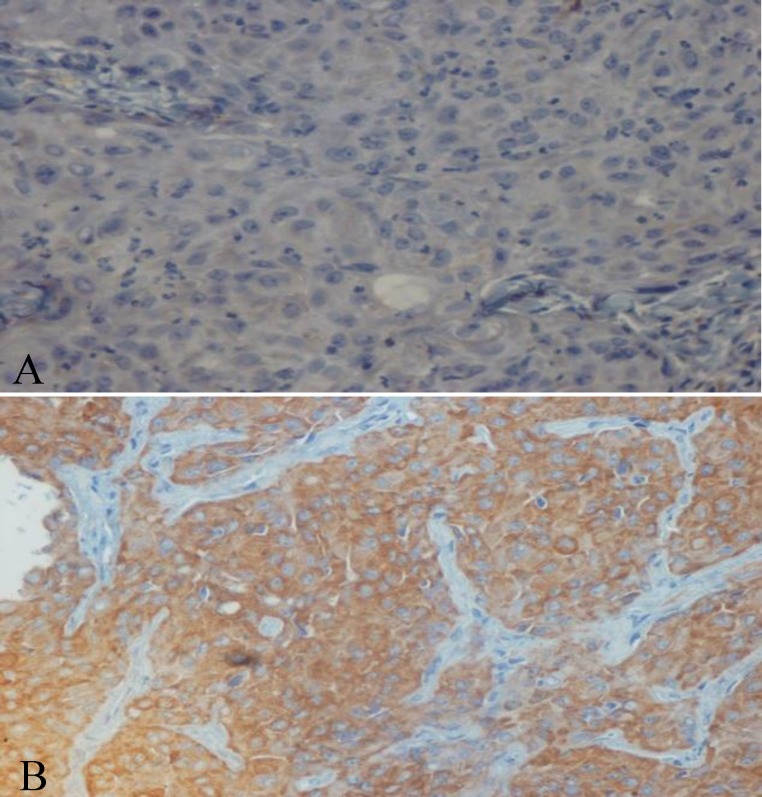
Immunostaining with CD_56_ in mucoepidermoid carcinoma (×40). A: negative staining of tumor cells in grade I. B: positive staining of tumor cells in grade III

Immunohistochemical expression of CD_56_ and ALDH_1_ markers in benign and malignant low-grade and high-grade salivary gland neoplasms are shown in Tables 2 and 3, respectively (Fig.1 to Fig.6).

**Fig 2:**
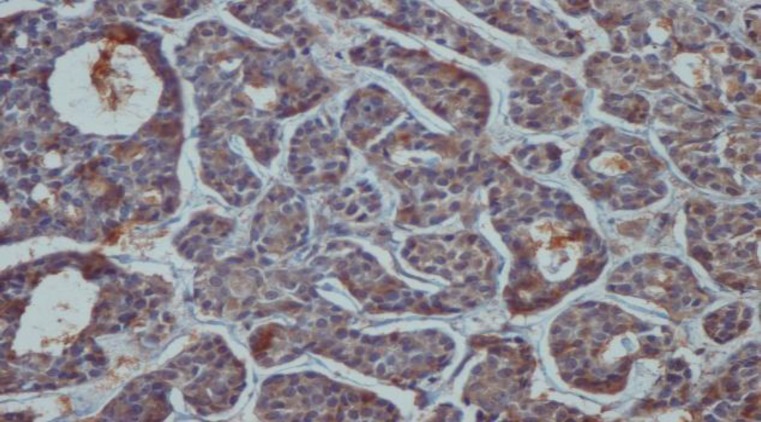
Positive immunohistochemical staining of tumor cells in solid adenoid cystic carcinoma with CD56 (×40

**Fig 3:**
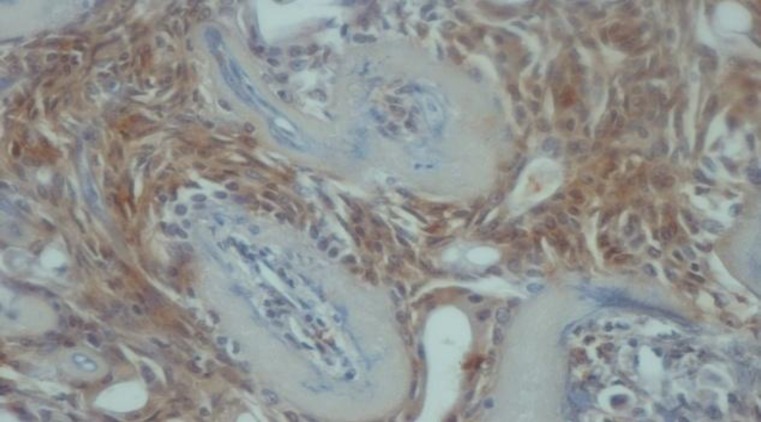
Positive immunohistochemical staining of tumor cells in pleomorphic adenoma with CD56 (×40)

**Fig4:**
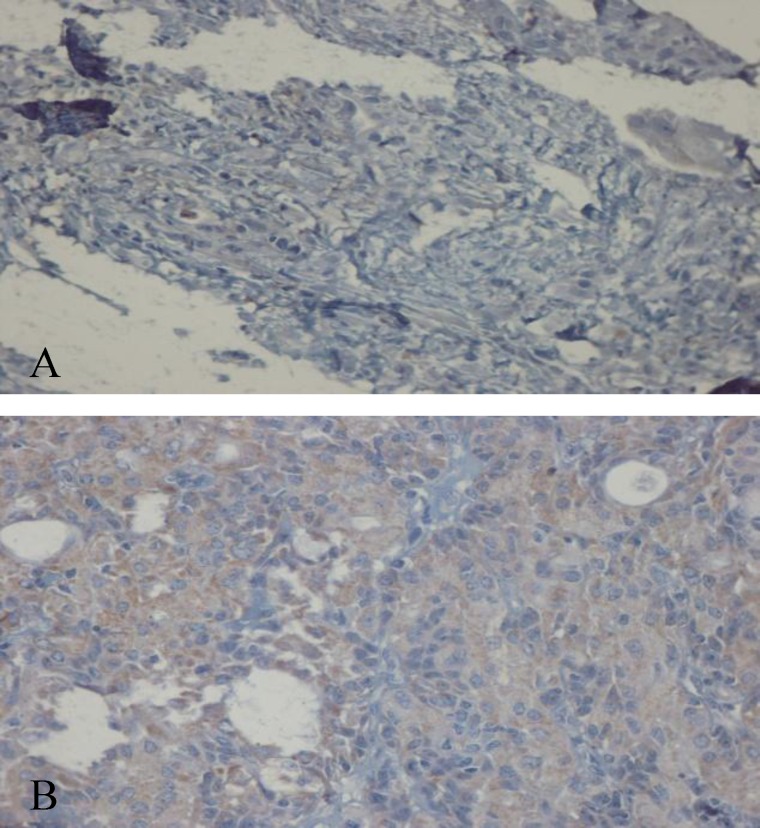
Immunostaining with ALDH1 in mucoepidermoid carcinoma (×40). A: negative staining of tumor cells in mucoepidermoid carcinoma grade III. B: positive staining of tumor cells in mucoepidermoid carcinoma grade I.

**Fig 5:**
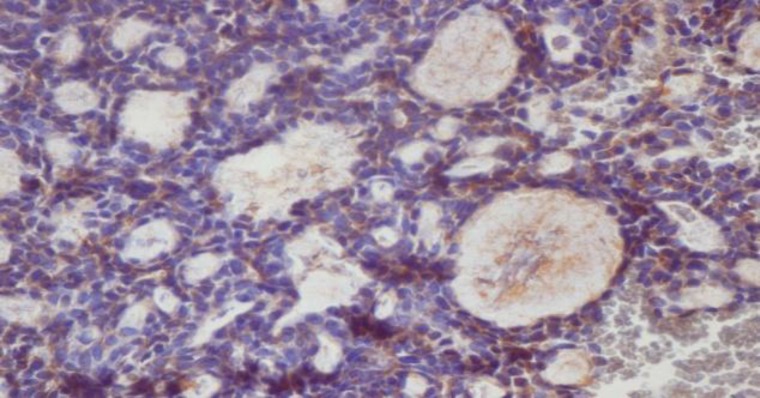
Positive immunohistochemical staining of tumor cells in Cribriform adenoid cystic carcinoma with ALDH_1_ (×40)

**Fig 6:**
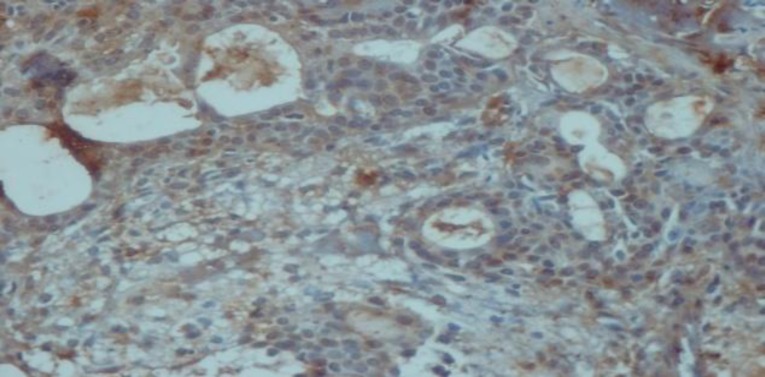
positive immunohistochemical staining of tumor cells in pleomorphic adenoma with ALDH 1 (×40)

According to Table 2, the differences in CD_56_ expression between benign and low-grade and high-grade malignant tumors were statistically significant (P=0.01). CD_56 _expression was higher in benign and high-grade malignant tumors. Also according to Table 2, no statistically significant difference in CD_56_ expression was observed between the mucoepidermoid carcinoma grade I and III (P=0.26). In addition, no statistically significant difference in terms of CD_56_ expression was observed between different histologic patterns of adenoid cystic carcinoma (cribriform, tubular, or solid patterns) (P=0.58). Finally, CD_56_ expression in salivary gland tumors showed no statistically significant relationship with location (major or minor salivary gland) (P=0.16), age (P=0.29), or gender (P=0.30).

According to Table 3, the difference between expression of ALDH_1_ in benign and low-grade and high-grade malignant salivary gland tumor was not statistically significant (P=0.54). 

Also according to Table 3, no statistically significant differences in expression of ALDH1 between mucoepidermoid carcinoma grade I and grade III was observed (P=0.43). In addition, there was no statistically significant difference in expression of ALDH1 among the different histologic patterns of adenoid cystic carcinoma (cribriform, tubular, or solid pattern) (P=0.54). 

Finally, ALDH_1 _expression in salivary gland tumors showed no statistically significant relationship with location (major or minor salivary gland) (P=0.20), age (P=0.58), or gender (P=0.27).

**Table 2:** Immunohistochemical expression of CD_56_ and ALDH_1_ in benign and malignant Low-grade and high-grade salivary gland neoplasms

**Tumor types**		_56_ ** expression ** **CD**	
		Negative	Positive
Pleomorphic adenoma		6 cases (40%)	9cases (60%)
Low-grade malignant tumors (Mucoepidermoid carcinoma grade I)		8 cases (100%)	0 case (0%)
high-grade malignant tumors	Mucoepidermoid carcinoma grade III	6 cases (27.3%)	1 case (4.5%)
	Adenoid cystic carcinoma	3 cases (13.6%)	12 cases (54.5%)

**Table 3:** Immunohistochemical expression of ALDH_1_ in benign and Low-grade and high-grade malignant salivary gland tumors

**Tumor types**		**Frequency based on the percentage of stained cells (P)**						**frequency based on intensity of staining(I** **(**				**Total score** **)** **P+I** **(**	
		**p=0**	**p=1**	**p=2**	**p=3**	**p=4**	**p=5**	**No staining** **(I=0)**	**week** **(I=1)**	**moderate** **(I=2)**	**intense** **(I=3)**	**Negative** **(0-2)**	**Positive** **(3-8)**
Pleomorphic adenoma		1	3	1	2	6	2	1	6	8	0	4cases (26.7%)	11cases (73.3%)
Low-grade malignant tumors (Mucoepidermoid carcinoma grade I)		1	0	4	1	1	1	1	7	0	0	1 case (12.5%)	7 cases (87.5%)
high-grade malignant tumors	Mucoepidermoid carcinoma grade III	2	0	3	0	2	0	2	4	1	0	2 cases (9%)	0 case (22.7%)
	Adenoid cystic carcinoma	0	1	1	5	6	2	0	4	10	1	1 case (4.5%)	14 case (63.6%)

Also in this study, the relationship between positive expression of ALDH_1_ and CD_56_ with each other was investigated and it was found that there was no statistically significant association between these two markers in salivary gland tumors (P=0.13).

## Discussion

The results of this study on salivary gland tumors indicate that expression of CD_56_, as an indicator of NK cells, is significantly higher in benign tumors and also high-grade malignant tumors. Based on these findings, it appears that a high density of NK cells in benign salivary pleomorphic adenoma represents the host immune system activity against cell damage. NK cells are the first line of the immune system response against harmful factors, but the number and function of NK cells and types (CD_56_^+ ^and CD_57_^+^) varies depending on the type of tumor and its stroma and grade in salivary gland tumors. Also according to the results of this study, it was observed that the number of CD_56_+ NK cells declined in the early stages of malignant salivary gland tumors formation. In order to justify these findings, we can say that probably in the long-term vicinity of NK cells with tumor antigens, a kind of compatibility between immune cells and tumor stroma, and NK cell sensitivity to tumor cells has been lost. Also, secretion factors such as TGF-β1, may cause a reduction in the number of CD_56_+ NK cells and an increase in NK cell inactive receptors. On the other hand, a decrease in the number of CD_56_+ NK cells may occur in parallel with an increase in other types of NK cell. However, with increasing tumor grade, malfunction of the normal NK cells occurs, meaning that the number of cytotoxic NK cells increases in the salivary gland tumors. Thus, an increase in cytotoxic NK cells is associated with tumor progression, high grade of malignancy, and metastasis in malignant salivary gland tumors.

Previous studies have reported controversial and conflicting results on the diagnostic and prognostic value of CD_56_. So far, few studies have investigated the expression of CD_56 _in salivary gland tumors. Nakatsuka and colleagues reported a case of invasive adenocarcinoma in the accessory parotid gland that showed positive expression of CD_56_ in a scattered pattern, although they used CD_56_ as a neuroendocrine marker (14). Weissferdt and Moran also reported five cases of rare salivary gland-type pulmonary tumors that showed characteristics of malignant mixed tumors, with neoplastic cells of these tumors showing positive staining for CD_56_ (15). Also Dutsch and colleagues evaluated the immunohistochemical expression of CD_56_ in parotid adenocarcinomas, and reported that 14% of the parotid adenocarcinomas were found to be CD_56_ positive. These researchers assumed CD_56_ as a marker of T lymphocytes that affect the performance of cytotoxic T cells (16). However, in the present study, approximately 43% of malignant tumors demonstrated positive expression for CD_56_.

In a study on breast carcinoma, Wachter and colleagues reported that CD_56_ expression was high in basal-like and luminal A-like breast carcinomas (17). Also in the study by Kontogianni and colleagues on 20 samples of small cell lung carcinoma, they reported that CD_56_ could be a valuable tool in the diagnosis of these cancers (18). Alegretti and colleagues reported that CD_56_ expression in acute myeloid leukemia is related to poor prognosis and lower survival rate (19). In addition, Aloysius and colleagues demonstrated that in pancreatic cancer samples, CD_56_ expression is associated with neural invasion and reduced survival (20), a finding that is consistent with the results of the present study. In a study on breast cancer, Mamessier and colleagues reported that aggressive types of disease have a reduced number of NK cell activating receptors such as CD_56_. These researchers suggested that factors such as TGF-β1 could play an important role in the reduced function of NK cells in tumors (21), contradicting the results of the present study.

In the present study, overexpression of ALDH_1_, as one of the most commonly used markers to detect cancer stem cells, was observed in all groups of salivary gland tumors, showing its role in tumorogenesis of salivary gland neoplasms. Recent empirical evidence suggests the existence of a tumorigenic population of cancer cells that demonstrate stem cell-like properties such as self-renewal and multipotency. These cells are able to both initiate and maintain tumor formation and progression of human cancers, such as salivary gland tumors (22).

In the present study, ALDH_1_ expression had no association with grade of mucoepidermoid carcinoma or histopathologic pattern of adenoid cystic carcinoma. This suggests that other factors and possibly other isoforms of ALDH, along with cancer stem cells, may play an important role in morphogenesis, cell differentiation, and development of salivary gland tumors.

In the study by Zhou and colleagues, ALDH_1_ expression in salivary adenoid cystic carcinoma was investigated in three groups: stromal staining, both stromal and epithelial staining, and no stromal or epithelial staining. These researchers demonstrated that the pattern of ALDH_1_ expression had no relation to histology, size, neural invasion, or survival of patients (23).

These findings are consistent with the results of the present study.

In a study by Fujita and Ikeda on salivary adenoid cystic carcinoma, it was suggested that the presence of small populations of cancer stem-like cells may play an important role in tumor morphogenesis through stimulating the extracellular matrix (24); in contrast with the results of the present study. A probable reason for this difference is that Fujita and Ikeda used CD_44_ and CD_133_ as the marker to identify cancer stem cells, while we used ALDH_1_. Therefore, based on the present study, ALDH_1_+ cancer stem cells appear to have no role in the morphogenesis of solid, tubular, and cribriform adenoid cystic carcinomas. However, perhaps the extracellular matrix, through CD_133_+ and CD_44_+ stem cells, plays an important role in regulating cell morphogenesis and various histologic patterns in adenoid cystic carcinoma.

Adams and colleagues demonstrated that salivary gland mucoepidermoid carcinomas contain a small population of cancer stem cells with enhanced tumorigenic potential and are characterized by high ALDH activity and CD_44_ expression. These results suggest that patients with mucoepidermoid carcinoma might benefit from therapies that ablate these highly tumorigenic cells (25), a finding which is consistent with the results of the present study. In a study on an isolated side population from the salivary gland mucoepidermoid carcinoma cell line MC3, Zhang and colleagues observed that sphere-forming assays could enrich stem cell-like cells, exhibiting high cloning efficiency and possessing strong tumorigenic ability (26), supporting the results of the present study.

In our study, we anticipate that pleomorphic adenomas that show overexpression of ALDH_1_ are more susceptible to malignant transformation. Indeed, Huang and colleagues demonstrated that ALDH_1_ overexpression in tongue squamous cell carcinoma was associated with metastasis and aggressive behavior (27).

Liu and colleagues investigated the role of ALDH_1_ in ovarian cancer and reported that overexpression of ALDH_1_ was associated with poor prognosis (28). Qiu and colleagues discussed five isoforms of 19 family members of ALDH in breast cancer and reported that all five isoforms can be found in different amounts in the tumoral tissues, but only the ALDH_1_ isoform is significantly associated with distant metastasis and patient survival. Thus only this isoform can be used as a poor prognostic indicator (29). In the present study, benign and malignant salivary gland tumors showed no statistically significant difference between expressions of ALDH_1_. In contrast, Schwartz and colleagues reported that benign breast tumors showed higher ALDH_1_ expression compared with malignant breast tumors (30).

Based on various studies, it appears that the ALDH family has 19 isoforms that are expressed to a greater extent in the cancer of specific areas of the body; for example ALDH_1_A_3_ isoform in breast cancer, ALDH_7_A_1_ isoform in prostate cancer, and ALDH_1_A_1_ in pancreatic cancer (31). Therefore, further studies are needed to elucidate the role of other possible ALDH isoforms in salivary gland tumors to complete the results of the present study.

In conclusion, it seems that cancer stem cells, based on the type of tumor and its stroma, may express several markers, such as those seen in lung cancer, with adenocarcinomas showing CD_133_ overexpression and squamous cell carcinomas showing ALDH_1_ overexpression (32). No significant relationship between expression of CD_56_ and ALDH_1_ markers with gender, age of patient, or location of tumor were found. An increased number of specimens may allow us to offer more accurate results.

## Conclusion

NK cells, which show high expression in benign and high-grade malignant salivary gland tumors, are important components of the anti-tumor immune response. Thus dysfunction of these cells may lead to tumor progression.

In the present study, overexpression of ALDH_1_, one of the most commonly used markers for the detection of cancer stem cells, was observed in all salivary gland tumors, which indicates the role of these cells in tumorogenesis of salivary gland neoplasms. On the other hand, a lack of correlation between ALDH_1_ expression and grade of differentiation or histopathologic pattern in malignant salivary gland tumors indicates that cancer stem cells might not have a role in the morphogenesis of salivary gland tumors, or that other isoforms of ALDH or other markers of cancer stem cells are effective in this regard. The results of the present study could be useful in the improvement of molecular-targeted therapies in salivary gland tumors.

## References

[1] Toshitaka N, Eiichi S, Rie I, Hisashi O, Reisuke H, Takeshi N (2012). Immunohistochemical Analysis of Salivary Gland Tumors: Application for Surgical Pathology Practice. Acta Histochem Cytochem.

[2] Neville BW, Dam DD, Allen CM, Bouquot JE (2016). Oral and maxillofacial pathology.

[3] Cheng M, Chen Y, Xiao W, Sun R, Tian Z (2013). NK cell-based immunotherapy for malignant diseases. Cellular & Molecular Immunology.

[4] Levy E, Roberti M, Mordoh J (2011). Natural Killer Cells in Human Cancer From Biological Functions to Clinical Applications. Journal of Biomedicine and Biotechnology.

[5] Miller J (2013). Therapeutic applications: natural killer cells in the clinic. American Society of Hematology.

[6] Ginestier C, Hur MH, Charafe-Jauffret E, Monville F, Dutcher J, Brown M et al (2007). ALDH1 is a marker of normal and malignant human mammary stem cells and a predictor of poor clinical outcome. Cell Stem Cell.

[7] Kakarala M, Wicha MS (2008). Implications of the Cancer Stem-Cell Hypothesis for Breast Cancer Prevention and Therapy. J Clin Oncol.

[8] Wang Y, Zhe H, Gao P, Zhang N, Li G, Qin J (2012). Cancer stem cell marker ALDH1 expression is associated with lymph node metastasis and poor survival in esophageal squamous cell carcinoma: a study from high incidence area of northern China. Dis Esophaqus.

[9] Michifuri Y, Hirohashi Y, Torigoe T, Miyazaki A, Kobayashi J, Sasaki T et al (2012). High expression of ALDH1 and SOX2 diffuse staining pattern of oral squamous cell carcinomas correlates to lymph node metastasis. Int Pathology.

[10] Brandwein MS, Ferlito A, Bradley PJ, Hille JJ, Rinaldo A (2002). Diagnosis and classification of salivary neoplasms: pathologic challenges and relevance to clinical outcomes. Acta Otolaryngol.

[11] Regezi J, Sciubba J, Jodan R, 6th ed. (2008). Oral Pathology, clinical Pathologic correlations.

[12] Demellawy D, Nasr AL, Babay S, Alowami S (2009). Diagnostic utility of CD56 immunohistochemistry in papillary carcinoma of the thyroid. Pathol Res Pract.

[13] Yan Q, Tianjie P, Li L, Fei C, Changli L, Linyong S (2014). The Expression of Aldehyde Dehydrogenase Family in Breast Cancer. J Breast Cancer.

[14] Nakatsuka S, Harada H, Fujiyama H, Takeda K, Kitamura K, Kimura H (2011). An invasive adenocarcinoma of the accessory parotid gland: a rare example developing from a low-grade cribriform cystadenocarcinoma. Diagnostic Pathology.

[15] Weissferdt A, Moran C (2011). Pulmonary salivary gland-type tumors with features of malignant mixed tumor (carcinoma ex pleomorphic adenoma): a clinicopathologic study of five cases. Am J Clin Pathol.

[16] Dutsch M, Lazar A, Tomaszewska R (2010). The Potential Role of MT and Vimentin Immunoreactivity in the Remodeling of the Microenvironment of Parotid Adenocarcinoma. Cancer Microenviron.

[17] Wachter D, Hartmann A, Beckmann M, Fasching P, Hein A, Bayer C (2014). Expression of Neuroendocrine Markers in Different Molecular Subtypes of Breast Carcinoma. BioMed Research International.

[18] Kontogianni K, Nicholson A, Butcher D, Sheppard M (2005). CD56: a useful tool for the diagnosis of small cell lung carcinomas on biopsies with extensive crush artifact. J Clin Pathol.

[19] Alegretti A, Bittar C, Bittencourt R, Piccoli A, Schneider L, Silla L (2011). The expression of CD56 antigen is associated with poor prognosis in patients with acute myeloid leukemia. Rev Bras Hematol Hemoter.

[20] Aloysius MM, Zaitoun AM, Awad S, Ilyas M, Rowlands BJ, Lobo DN (2010). Mucins and CD56 as markers of tumour invasion and prognosis in periampullary cancer. Br J Surg.

[21] Mamessier E, Sylvain A, Thibult M, Houvenaeghel G, Jacquemier J, Castellano R (2011). Human breast cancer cells enhance self-tolerance by promoting evasion from NK cell antitumor immunity. The Journal of Clinical Investigation.

[22] Adams A, Warner K, Nör J-E (2013). Salivary Gland Cancer Stem Cells. Oral Oncol.

[23] Zhou J, Hanna E, Roberts D, Weber R, Bell D (2013). ALDH1 immunohistochemical expression and its significance in salivary adenoid cystic carcinoma. Journal of the Science and Specialties of Head and Neck.

[24] Fujita S, Ikeda T (2012). Cancer stem-like cells in adenoid cystic carcinoma of salivary glands: relationship with morphogenesis of histological variants. Journal of Oral Pathology & Medicine.

[25] Adams A, Warner K, Pearson A, Zhang Z, Kim H, Mochizuki D (2015). ALDH/CD44 identifies uniquely tumorigenic cancer stem cells in salivary gland mucoepidermoid carcinomas. Oncotarget.

[26] Zhang L, Xia Y, Li L, Wang Y, Liu Y, Li C (2012). Cancer stem cell-like cells exist in mucoepidermoid carcinoma cell line MC3. Oncol Res.

[27] Huang CF, Xu XR, Wu TF, Sun ZJ, Zhang WF (2014). Correlation of ALDH1, CD44, OCT4 and SOX2 in tongue squamous cell carcinoma and their association with disease progression and prognosis. J Oral pathol Med.

[28] Liu S, Liu C, Min X, Ji Y, Wang N, Liu D (2013). Prognostic Value of Cancer Stem Cell Marker Aldehyde Dehydrogenase in Ovarian Cancer: A Meta-Analysis. Plos one.

[29] Qiu Y, Pu T, Li L, Cheng F, Lu C, Sun L (2014). The Expression of Aldehyde Dehydrogenase Family in Breast Cancer. Journal of Breast Cancer.

[30] Schwartz T, Stark A, Pang J, Awuah B, Kleer CG, Quayson S et al (2013). Expression of aldehyde dehydrogenase 1 as a marker of mammary stem cells in benign and malignant breast lesions of Ghanaian women. Cancer.

[31] Marcato P, Dean CA, Giacomantonio CA, Lee PW (2011). Aldehyde dehydrogenase: its role as a cancer stem cell marker comes down to the specific isoform. Cell Cycle.

[32] Gonzalez A, Salas C, Provencio M, Orcajo N, Diez-Tascon C, Santin E (2015). The role of ALDH1 and CD133 expression as stem cell tumor marker in early-stage NSCLC. J Clin Oncol.

